# Impaired lipid homeostasis and elevated lipid oxidation of erythrocyte membrane in adolescent depression

**DOI:** 10.1016/j.redox.2025.103491

**Published:** 2025-01-08

**Authors:** Jinfeng Wang, Xiaowen Hu, Ya Li, Shuhui Li, Tianqi Wang, Dandan Wang, Yan Gao, Qian Wang, Jiansong Zhou, Chunling Wan

**Affiliations:** a Bio-X Institutes, Key Laboratory for the Genetics of Developmental and Neuropsychiatric Disorders, Ministry of Education, Shanghai Jiao Tong University, Shanghai, China; b School of Rehabilitation Medicine, Shandong Second Medical University, Weifang, Shandong, China; c Department of Psychiatry, National Clinical Research Center for Mental Disorders, and National Center for Mental Disorders, The Second Xiangya Hospital of Central South University, Changsha, Hunan, China; d Shanghai Mental Health Center, Shanghai Key Laboratory of Psychiatry Disorders, Shanghai Jiao Tong University, Shanghai, China

**Keywords:** Adolescent depression, Lipidome, Membrane fluidity, Oxidative stress, Cognitive function, Niacin skin flushing response

## Abstract

Adolescent depression is a globally concerned mental health issue, the pathophysiological mechanisms of which remain elusive. Membrane lipids play a crucial role in brain development and function, potentially serving as a crossroad for the abnormalities in neurotransmitters, neuroendocrine, inflammation, oxidative stress, and energy metabolism observed in depressed adolescents. The primary aim of this study was to investigate the erythrocyte membrane lipid profile in adolescent depression. A total of 2838 erythrocyte membrane lipids were detected and quantified in 81 adolescents with depression and 67 matched healthy adolescents using ultra-high performance liquid chromatography-mass spectrometry. Depressed adolescents exhibited significantly different membrane lipid characteristics compared to healthy controls. Specifically, the levels of cholesterol, sphingomyelins, and ceramides were increased, while ether lipids were decreased in patients. Moreover, the patients showed reduced polyunsaturated fatty acids and elevated lipophilic index in membrane, suggesting diminished membrane fluidity. The higher oxidized membrane lipids and plasma malondialdehyde were observed in adolescent depression, indicating the presence of oxidative stress. Importantly, membrane lipid damage was associated with more severe depressive symptoms and worse cognitive function in patients. In addition, reduced polyunsaturated fatty acids and membrane fluidity may be partly responsible for the blunted niacin skin flushing response found in depressed adolescents. In conclusion, our results reveal impaired erythrocyte membrane lipid homeostasis in adolescents with depression, which may implicate membrane dysfunction in the brain. These findings offer new insights into the underlying molecular mechanisms of adolescent depression, highlighting the potential of counteracting membrane damage as a promising avenue for future therapeutic interventions.

## Highlights

• Altered membrane lipidome may be involved in the pathophysiology of adolescent depression.
• Polyunsaturated fatty acid levels and membrane fluidity were reduced in patients.
• Membrane lipid peroxidation was increased, indicating elevated oxidative stress.
• Elevated cholesterol, sphingolipid, and ceramide levels may affect lipid rafts' function.
• Disturbed membrane lipids were related to depressive symptoms and cognitive impairment.

## Graphical abstract

**Figure:**
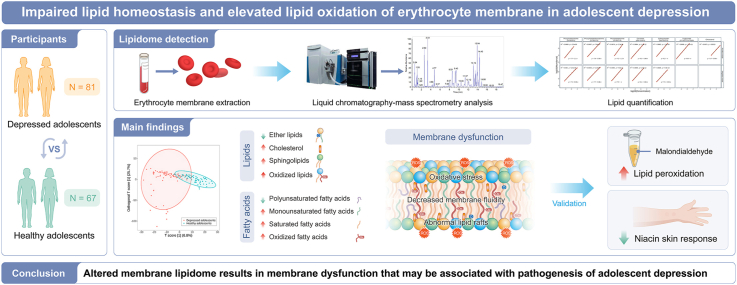

## 1 Introduction

Depressive disorder (DD) is a widespread mental illness that is considered the second leading cause of disability [1]. Adolescence is a particularly vulnerable period for depression with a global prevalence of 8 % [2], and 25.2 % adolescents were experiencing clinically elevated depression symptoms during the COVID-19 epidemic [3]. Adolescent depression exhibits clinical symptoms that significantly impact the academic performance and physical health, such as weight change, energy loss, insomnia, and cognitive impairment [4]. However, the pathophysiology and etiology of adolescent depression remain insufficiently understood, which limits the effectiveness of current antidepressants for this population [5].

Adolescents with depression manifest as multi-system abnormalities involving neurotransmitters, neuroendocrine, inflammatory response, oxidative stress, and energy metabolism. It is hypothesized that the underlying physiological basis linking these pathophysiological processes is associated with membrane lipids [6]. The membrane lipids significantly influence the membrane physical characteristics, membrane proteins functions, and lipids-proteins interactions [7]. Polyunsaturated fatty acids (PUFA) in lipids can reduce membrane bending rigidity, increase membrane fluidity, and facilitate cellular processes involving membrane deformation, such as synaptic vesicle activities [8]. PUFAs are highly susceptible to oxidation, and lipid peroxidation induced by oxidative stress can lead to atypical cell death [9]. Cholesterol and sphingolipids (SP) form lipid raft domains through hydrogen bonding, playing a role in neurotransmitter signaling [10]. Certain lipids serve as first and second messengers in signal transduction, such as eicosanoids derived from membrane arachidonic acid (AA) and eicosapentaenoic acid (EPA), contributing to the maintenance of inflammatory balance [11]. Moreover, lipids are vital energy sources. It is widely recognized that deviations in neuronal lipid composition are linked to anomalous neurodevelopment [12]. Research suggests that chronic severe stress can cause oxidative damage to membrane lipids, leading to abnormalities in neurotransmitter signaling and information processing in synapses and neural circuits that mediate emotional and cognitive functions, which may be involved in the pathological mechanism of adolescent depression [13].

Abnormal lipid composition has been found in both central and peripheral systems in adult patients with depression. In the brain, several studies have reported abnormal levels of phosphomonoesters and phosphodiesters in depression, indicating disrupted phospholipid metabolism [[14], [15], [16]]. In the peripheral blood, meta-analysis has confirmed the diminished levels of n3 PUFA in depression, which was potentially attributed to oxidative damage [17,18], and an imbalance between n6 PUFA and n3 PUFA was repeatedly verified [19,20]. It is worth noting that the pathophysiological mechanisms of adolescent depression may differ from those of adult depression, particularly considering that adolescents are in a developmental stage [5,21,22]. A few studies have observed abnormal fatty acid composition of erythrocyte membranes in adolescent depression with mixed results [[23], [24], [25], [26]]. In addition, the diverse arrangement of different head groups and fatty acyl chains in glycerophospholipids (GP) and SP results in a wide variety of lipid species [27]. However, previous investigations on membrane lipids predominantly report overall fatty acid levels with individual lipid changes neglected, which means that little is known about the variations in membrane lipids with different head groups. Therefore, a comprehensive analysis of the cell membrane lipid composition in adolescents with depression is imperative.

Additionally, our previous study revealed a notable decrease in the niacin skin flushing response (NSFR) among adolescents with depression, which may be a potential auxiliary diagnostic marker [28]. The underlying molecular mechanism of NSFR involves membrane AA metabolism. After niacin stimulation of the skin, it binds to hydroxycarboxylic acid receptor 2 (HCAR2) on skin keratinocytes or Langerhans cells [29,30], subsequently stimulating phospholipase A2 (PLA2) activity to catalyze the hydrolysis of AA from membrane [31]. AA is then metabolized by cyclooxygenase 2 (COX2) to prostaglandin E2 (PGE2) and D2 (PGD2), which stimulate vasodilation and trigger the skin flushing response [32]. Previous studies have shown that the abnormalities of NSFR in schizophrenia are related to the dysregulation of membrane AA metabolism [33,34]. We speculate that the blunted NSFR in adolescent depression may indicate a disturbance of membrane lipid homeostasis as well.

The primary objective of this study was to investigate the erythrocyte membrane lipid profile in adolescent depression and identify the specific membrane lipid characteristics associated with the disease. Secondly, we aimed to further explore whether the membrane lipid homeostasis was related to the depressive symptom and cognitive function of patients. Finally, we attempted to establish a link between membrane lipid homeostasis and NSFR, with the aim to at least partially understand the molecular mechanisms underlying the diminished NSFR in adolescent depression.

## 2 Material and methods

### 2.1 Study participants

Eighty-one adolescents aged 13 to 24 years with a diagnosis of DD according to the International Classification of Diseases, Version 10 (ICD-10) criteria were recruited from the Fourth People's Hospital of Wuhu in Anhui Province, China. All patients were never medicated or have undergone a drug washout period of at least two weeks upon enrollment. The severity of depressive symptoms was assessed using the Hamilton Depression Scale (HAMD), while cognitive function was evaluated using the Montreal Cognitive Assessment (MoCA) and the Wechsler Memory Scale (WMS). Sixty-seven healthy adolescents matched in terms of age, sex, and body mass index (BMI) were recruited. Detailed information on the demographic and clinical characteristics of depressed adolescents and healthy controls (HC) can be found in Table 1. Written informed consent was obtained from all participants, with parental consent provided for participants under 18 years of age. This study was approved by the local ethics committee (No. 2020-KY-15) and carried out in an accordance with the Helsinki Declaration.

**Table 1:** Demographic and clinical characteristics of DD and HC.

Variables	DD (N = 81)	HC (N = 67)	*p*-value
Male/Female ^1^	33/48	37/30	0.112
Age (years) ^2^	16.00 (3.50)	18.00 (3.00)	0.080
Height (cm) ^2^	167.00 (13.00)	168.00 (12.75)	0.994
Weight (kg) ^2^	60.00 (20.00)	58.00 (15.00)	0.266
BMI (kg/m^2^) ^2^	20.90 (4.57)	20.76 (4.36)	0.193
HAMD scores	27.0 (7.50)	NA	/
MoCA scores	28.00 (3.00)	NA	/
WMS scores	101.00 (29.00)	NA	/

Continuous variables are represented by median with interquartile range (IQR). ^1^ Analyzed by the Chi-square test. ^2^ Analyzed by Mann-Whitney *U* test. DD, Depressive Disorder; HC, Healthy Control; HAMD, Hamilton Depression Scale; MoCA, Montreal Cognitive Assessment; WMS, Wechsler Memory Scale; BMI, Body Mass Index.

### 2.2 Sample collection and preparation

Venous blood was collected from all participants after overnight fasting. The red blood cells were separated by centrifugation at 1600*g* for 10 min at 4 °C and stored at −80 °C until use.

100 μL erythrocytes were suspended overnight with 2 mL Tris-HCL (pH = 7.4, 10 mM) until swelling and bursting. The supernatant was removed by centrifugation, and the precipitated erythrocyte membranes were washed three times with PBS. The erythrocyte membrane lipids were then extracted with 300 μL of methanol/chloroform mixtures (1:2, v/v, containing 0.83 μg/mL cardiolipin as an internal standard) at 40 °C for 2 h. After drying, the lipids were dissolved in 100 μL of dichloromethane/isopropanol/methanol mixtures (1:1:2, v/v/v). Quality control (QC) samples were prepared by pooling and mixing equal aliquots of each sample.

### 2.3 UPLC-QEMS analysis

Lipidomic analysis was performed by the Vanquish UHPLC system & Q Exactive plus Mass spectrometer (Thermo Fisher Scientific, MA, USA) with a BEH C18 column (100 ∗ 2.1 mm, 1.7 μm, Waters, MA, USA) in both positive and negative modes. The mobile phase A was acetonitrile/water (6:4, v/v) with 10 mM ammonium formate and 0.1 % formic acid (v/v), and mobile phase B was isopropanol/acetonitrile (9:1, v/v) with 10 mM ammonium formate and 0.1 % formic acid (v/v). The elution gradient ranged from 95/5 to 0/100 over 17 min, with maintaining the column temperature at 55 °C and a flow rate of 0.4 mL/min. The QEMS parameters were as follows: the scan mode was set to data-dependent acquisition (DDA) mode, consisting of one full scan followed by six MS/MS scans. The full scan range spanned from 150 to 2000 atomic mass units (amu), with a resolution of 70000. For dd-MS/MS scans, the resolution was set to 17500. The spray voltages were adjusted to 3.8 kV for positive mode and 3.0 kV for negative mode, while the capillary temperature was maintained at 320 °C.

Data were processed by Lipidsearch 4.2 (Thermo Fisher Scientific, MA, USA) for peak picking and alignment to produce peak intensities for retention time and *m/z* data pairs. Compounds were identified based on accurate mass and fragments in MS-MS. Raw peak areas of all identified lipids were exported by LipidSearch 4.2 and normalized using QC samples [35] and the median of peak area to correct the sampling and systematic error. Seven standard substances were used for lipid quantification (Fig. S1), and lipid concentrations were expressed in molar units (μmol/L).

### 2.4 ELISA-based lipid peroxidation validation

Detection of malondialdehyde (MDA) was performed using an ELISA kit (Elabscience, Wuhan, China). The test was conducted according to the manufacturer's instructions. Briefly, 50 μL standard or plasma was mixed with 50 μL biotinylated detection antibody and incubate for 45 min at 37 °C. Aspirate and wash the plate for 3 times. Add 100 μL HRP conjugate and incubate for 30 min. After washing 5 times, 90 μL substrate reagent was added and incubate for 15 min. Add 50 μL stop solution and detect the OD values at 450 nm immediately.

### 2.5 Niacin skin test

The detection method of NSFR was consistent with the previously published article [28], using BrainAid Skin Niacin Response Test Instrument TY-AraSnap-H100 (Shanghai Tianyin Biological Technology Ltd., Shanghai, China). A patch containing 6 concentrations (triple gradient dilution starting at 60 mM) of aqueous methyl nicotinate (AMN, C7H7NO2, 99 %, Sigma-Aldrich, Darmstadt, Germany) was applied to the forearm for 1 min and then removed. Flushing response on the forearm was photographed at every 10 s for a duration of 10 min and was evaluated by intelligent image recognition software. In this study, the sum of flushing areas of 6 concentrations at the 10th minute was used as the index of NSFR.

### 2.6 Statistical analysis

Principal component analysis (PCA) and orthogonal partial least square discriminant analysis (OPLS-DA) were conducted for multivariate statistical analysis, and the variable importance in the projection (VIP) of each lipid was obtained. Lipids were compared between groups using Mann-Whitney *U* test, and p-values were corrected using false discovery rate (FDR) method. Lipids with VIP >1, FDR <0.05, and fold change (FC) > |1.2| were identified as differential lipids. Hypergeometric distribution was used for enrichment analysis. To identify potential diagnostic biomarkers for DD, classification performance was evaluated by logistic regression and area under the curve (AUC).

The concentration of each fatty acid was calculated as the sum of the concentrations of individual lipids containing the same acyl chain. The saturated fatty acid (SFA) level was determined by the sum of the content of fatty acids without double bonds, the monounsaturated fatty acid (MUFA) level was the sum of the content of fatty acids with one double bond, and the PUFA level was the sum of the content of fatty acids with two or more double bonds. The calculation of fatty acid indexes, including unsaturation index, chain length index, lipophilic index, and peroxidation index, followed the methodology outlined by Li et al. [34]. Specifically, unsaturation index was calculated by summing the products of each fatty acid proportion (as a percentage of the total fatty acid content) and the number of double bonds. Chain length index was calculated by summing the products of each fatty acid proportion and the number of carbon atoms. Lipophilic index was calculated as the sum of the products of each fatty acid proportion and its melting point (°C), and then dividing this sum by the total proportion of all fatty acids. Peroxidation index was calculated as follows: (% monoenoic acid ∗ 0.025) + (% dienoic acid ∗ 1) + (% trienoic acid ∗ 2) + (% tetraenoic acid ∗ 4) + (% pentaenoic acid ∗ 6) + (% hexaenoic acid ∗ 8). The differences between groups in fatty acid levels and fatty acid indexes were evaluated using Mann-Whitney *U* test.

The weighted correlation network analysis (WGCNA) and Spearman's rank coefficient were applied to explore the relationships between lipids and clinical symptoms, including HAMD, MoCA, WMS, and NSFR. A p-value <0.05 (or FDR <0.05) was considered statistically significant. R 4.3.1 was used for statistical analysis.

## 3 Results

### 3.1 Membrane lipid was abnormal in adolescent depression

**Fig. 1:**
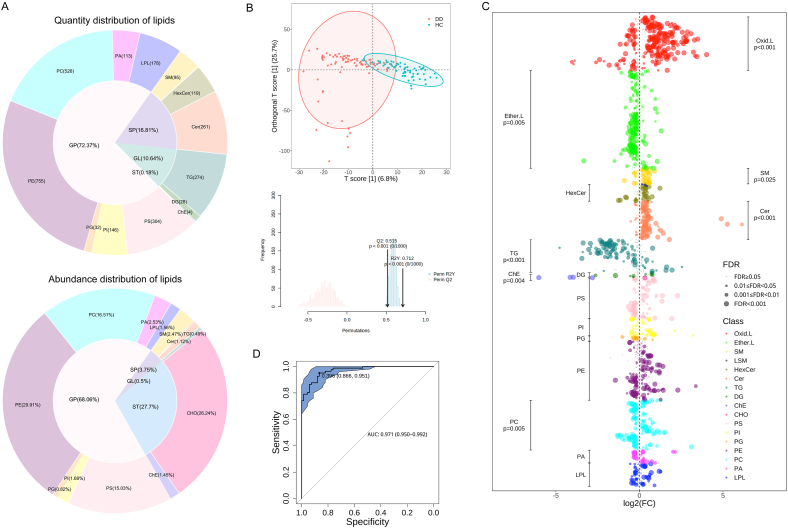
**Membrane lipid characteristics were significantly different between depressed adolescents and controls.** (A) Quantity and abundance distribution of different lipid classes in the erythrocyte membrane. (B) OPLS-DA model and permutation testing of erythrocyte membrane lipidome. (C) The differences in 2838 lipids between depressed adolescents and controls, in which the marked p-value was obtained by hypergeometric test. p<0.05 indicates that the lipid class is significantly increased (right) or decreased (left) in adolescent depression. (D) Diagnostic efficacy of the combinational biomarker panel of 8 lipids. DD, Depressive Disorder; HC, Healthy Control; OPLS-DA, Orthogonal Partial Least Squares-Discriminant Analysis; GP, Glycerophospholipid; ST, Sterol Lipids; GL, Glycerolipid; SP, Sphingolipid; LPL, Lysophospholipid; PA, Phosphatidic Acid; PC, Phosphatidylcholine; PE, Phosphatidylethanolamine; PG, Phosphatidylglycerol; PI, Phosphatidylinositol; PS, Phosphatidylserine; CHO, Cholesterol; ChE, Cholesterol Ester; DG, Diglyceride; TG, Triglyceride; Cer, Ceramide; HexCer, Hexosyl Ceramide; SM, Sphingomyelin; LSM, Lysosphingomyelin; Ether.L, Ether Lipid; Oxid.L, Oxidized Lipid; FC, Fold Change; FDR, False Discovery Rate; AUC, Area Under the Curve.

A total of 2838 erythrocyte membrane lipids were detected and quantified (Fig. 1A), which were categorized into 15 classes, including phosphatidic acid (PA), phosphatidylcholine (PC), phosphatidylethanolamine (PE), phosphatidylglycerol (PG), phosphatidylinositol (PI), phosphatidylserine (PS), lysophospholipids (LPL), cholesterol ester (ChE), cholesterol (CHO), diglyceride (DG), triglyceride (TG), ceramide (Cer), hexosyl ceramide (HexCer), sphingomyelin (SM), and lysosphingomyelin (LSM). These 15 lipid classes were further grouped into GP, SP, sterol lipids (ST), and glycerolipids (GL). GP was the most abundant lipid category, accounting for 72.37 % in quantity and 68.06 % in abundance, especially PE and PC. SP accounted for 16.81 % of the quantity and 3.75 % of the abundance. Although ST accounted for only 0.18 % in quantity, its abundance comprised 27.7 % of the total lipid content. In contrast, GL represented 10.64 % of the total quantity, the abundance ratio of which was only 0.5 %.

PCA of the 2838 membrane lipids revealed a separation tendency between the DD and HC groups, with significant differences observed in PC1 (p = 0.014) and PC2 (p < 0.001) (Fig. S2). Further analysis using OPLS-DA confirmed significant distinctions in membrane lipid profile between the groups (R^2^Y: 0.712, Q^2^: 0.515), and 1000 times of permutation tests indicated no overfitting of the model (Fig. 1B). The distribution of FC and FDR of the 2838 lipids is shown in Fig. 1C, with oxidized lipids (Oxid.L) and ether lipids (Ether.L) classified separately due to their unique structure. Among these lipids, 651 species exhibited significant differences between the DD and HC groups (VIP >1 & FDR <0.05 & FC > |1.2|), with 293 downregulated and 358 upregulated in DD (Table S1). Enrichment analysis revealed that the significantly downregulated lipids in DD were mainly Ether.L (p = 0.005), PC (p = 0.005), ChE (p = 0.004), and TG (p < 0.001), while the significantly upregulated lipids were mainly Oxid.L (p < 0.001), Cer (p < 0.001), and SM (p = 0.025). Furthermore, we found that differential membrane lipids may help identify DD from HC. A model comprising 8 lipids demonstrated excellent diagnostic efficacy for adolescent depression (Table S2), with an AUC of 0.971 (sensitivity = 0.951, specificity = 0.866, Fig. 1D). Therefore, these results suggested that the membrane lipid profile of depressed adolescents was significantly different from that of healthy adolescents.

### 3.2 PUFA and membrane fluidity were decreased in adolescent depression

**Fig. 2:**
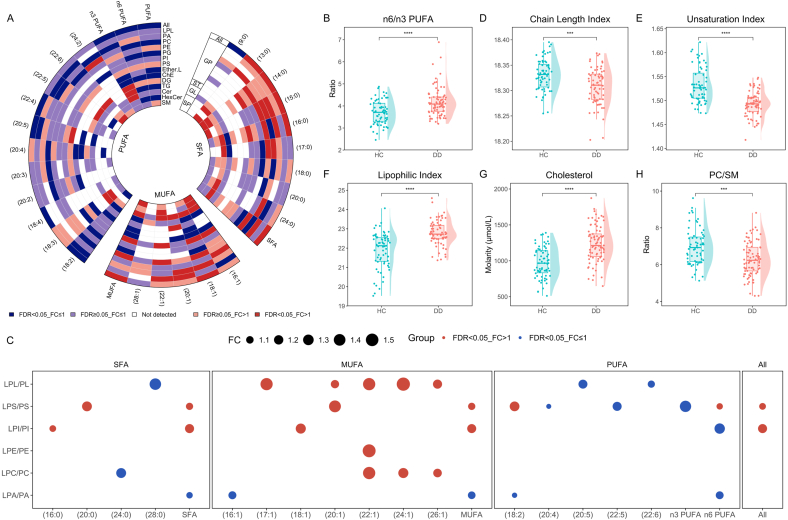
**PUFA and membrane fluidity were decreased in adolescent depression.** The difference of (A) fatty acids levels in different lipid classes, (B) n6/n3 PUFA ratio, (C) ratios of lysophospholipid to phospholipid, (D) chain length index, (E) unsaturation index, (F) lipophilic index, (G) cholesterol levels, and (H) PC/SM ratio between depressed adolescents and controls. ∗p<0.05, ∗∗p<0.01, ∗∗∗p<0.001, ∗∗∗∗p<0.0001. DD, Depressive Disorder; HC, Healthy Control; LPL, Lysophospholipid; PL, Phospholipid; LPA, Lysophosphatidic Acid; PA, Phosphatidic Acid; LPC, Lysophosphatidylcholine; PC, Phosphatidylcholine; LPE, Lysophosphatidylethanolamine; PE, Phosphatidylethanolamine; PG, Phosphatidylglycerol; LPI, Lysophosphatidylinositol; PI, Phosphatidylinositol; LPS, Lysophosphatidylserine; PS, Phosphatidylserine; Ether.L, Ether Lipid; ChE, Cholesterol Ester; DG, Diglyceride; TG, Triglyceride; Cer, Ceramide; HexCer, Hexosyl Ceramide; SM, Sphingomyelin; GP, Glycerophospholipid; ST, Sterol Lipids; GL, Glycerolipid; SP, Sphingolipid; SFA, Saturated Fatty Acids; MUFA, Monounsaturated Fatty Acids; PUFA, Polyunsaturated Fatty Acids; FC, Fold Change; FDR, False Discovery Rate.

Next, we conducted a detailed analysis of the alterations in membrane lipids from the perspective of fatty acid chain. Interestingly, within the GP category, lipids containing PUFA were predominantly decreased, while those containing SFA and MUFA were mostly increased, with the exception of Ether. L (Fig. S3). In addition, ST, GL, and SP showed collective trends within their respective classes irrespective of the fatty acid chains. Subsequently, we assessed the change of fatty acid content by adding up the molarity of lipids containing the same fatty acyl chain. As depicted in Fig. 2A, there was a notable elevation in the levels of SFA and MUFA in DD. Specifically, C16:0 and C18:1 with the higher abundance in membrane were increased significantly, especially in PA, PE, PS, and Cer. In contrast, PUFA levels showed a notable reduction, including C18:2, C22:4, C20:5 (EPA), and C18:4, especially in PC and Ether.L. The marked decrease of n3 PUFA and n6 PUFA were observed in DD, while the ratio of n6/n3 PUFA showed a significant increase (Fig. 2B), potentially compromising anti-inflammatory and antioxidant capacities. In addition, we adopted the ratio of lysophospholipid to phospholipid (LPL/PL) to indicate the flux of the hydrolysis from membrane PL to LPL. It was found that the ratios of LPL/PL containing PUFA were decreased while those containing MUFA or SFA mainly showed significant elevation in patients (Fig. 2C). This finding indicated that fewer LPL with PUFA tails remained in the membrane, and more hydrolysis of PUFA to the cytosol occurred.

The differences in fatty acid chains and lipid classes impact the erythrocyte membrane status. The chain length index (Fig. 2D) and unsaturation index (Fig. 2E) were notably reduced in depressed adolescents, which have an impact on membrane fluidity. The lipophilic index that is a comprehensive index of membrane fluidity was significantly increased in DD (Fig. 2F), indicating the attenuation of membrane fluidity. Simultaneously, the elevated cholesterol level (Fig. 2G) and decreased PC/SM ratio (Fig. 2H) in DD indicated an adverse effect on cell membrane fluidity as well. These results revealed the disturbance of membrane fatty acid composition and diminished membrane fluidity in adolescent depression.

### 3.3 Lipid oxidation was increased in adolescent depression

**Fig. 3:**
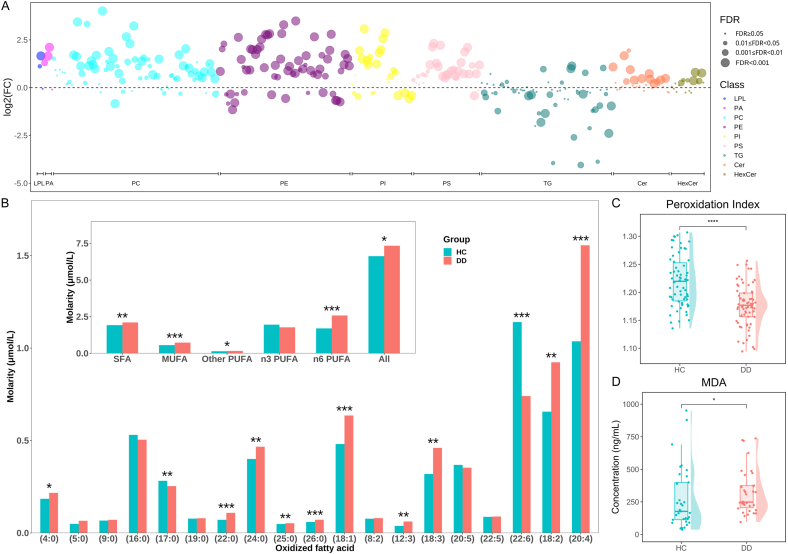
**Lipid oxidation was increased in adolescent depression.** The differences of (A) 324 oxidized lipids levels, (B) oxidized fatty acids levels (the inner diagram is the sum of different oxidized fatty acids), (C) peroxidation index, and (D) plasma MDA levels between depressed adolescents and controls. ∗p<0.05, ∗∗p<0.01, ∗∗∗p<0.001, ∗∗∗∗p<0.0001. DD, Depressive Disorder; HC, Healthy Control; LPL, Lysophospholipid; PA, Phosphatidic Acid; PC, Phosphatidylcholine; PE, Phosphatidylethanolamine; PI, Phosphatidylinositol; PS, Phosphatidylserine; TG, Triglyceride; Cer, Ceramide; HexCer, Hexosyl Ceramide; SFA, Saturated Fatty Acids; MUFA, Monounsaturated Fatty Acids; PUFA, Polyunsaturated Fatty Acids; MDA, Malondialdehyde; FC, Fold Change; FDR, False Discovery Rate.

As previously mentioned, lipid oxidation was increased in adolescents with depression (Fig. 1C). Of the 324 oxidized lipids detected, 186 showed significant differences between the DD and HC groups, of which 148 were significantly elevated in the DD group (Fig. 3A). These oxidized lipids were mainly derived from GP, especially PC, PE, PI, and PS. Fatty acid chains were the site of lipid peroxidation. Fig. 3B and Fig. S4 have shown the difference of the oxidized fatty acid chain levels between the DD and HC groups. Our study revealed a significant increase in the oxidation of n6 PUFA in the DD group, among which the oxidized C20:4 (AA) and C18:2 accounted for the highest proportion. The oxidation of SFA and MUFA were also notably elevated. Additionally, the peroxidation index calculated based on the double bonds of membrane fatty acid was observed to be significantly lower in the DD group (Fig. 3C), which represents a diminished ability to neutralize reactive oxygen species (ROS). To further determine the oxidation status, the plasma level of MDA, a lipid peroxidation product, was measured and observed to be elevated in DD (Fig. 3D). Overall, these findings suggested an escalation of oxidative stress in adolescents with depression, which caused lipid damage in cell membranes.

### 3.4 Impaired membrane lipids were associated with depressive symptoms and cognitive function

**Fig. 4:**
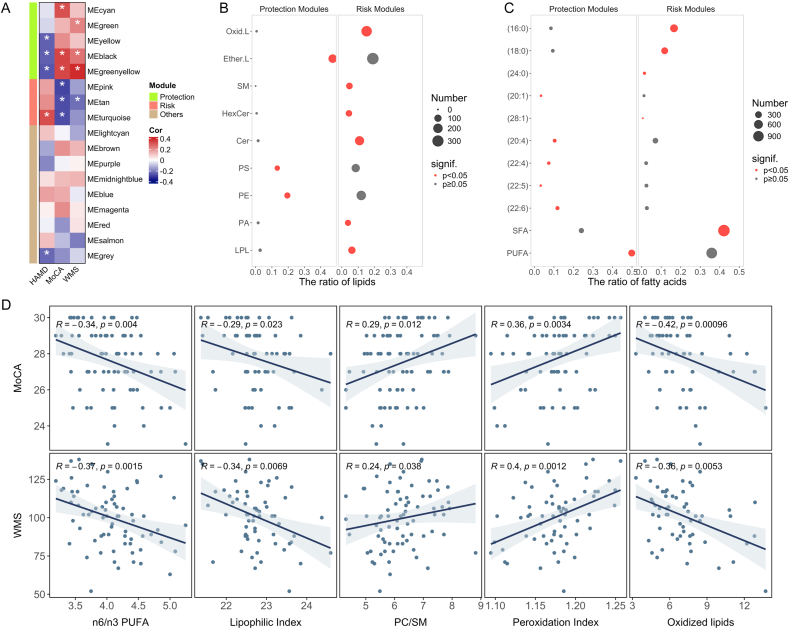
**Membrane lipid homeostasis was associated with depressive symptom and cognitive function of adolescents with depression.** (A) 8 lipid modules significantly associated with clinical symptoms. ∗p < 0.05. The composition of (B) lipid classes and (C) fatty acid chains in protection and risk modules. (D) The association between MoCA and WMS scores with n6/n3 PUFA ratio, lipophilic index, PC/SM ratio, peroxidation index, and total content of oxidized lipids. HAMD, Hamilton Depression Scale; MoCA, Montreal Cognitive Assessment; WMS, Wechsler Memory Scale; LPL, Lysophospholipid; PA, Phosphatidic Acid; PC, Phosphatidylcholine; PE, Phosphatidylethanolamine; PS, Phosphatidylserine; Cer, Ceramide; HexCer, Hexosyl Ceramide; SM, Sphingomyelin; Ether.L, Ether Lipid; Oxid.L, Oxidized Lipids; SFA, Saturated Fatty Acids; PUFA, Polyunsaturated Fatty Acids.

We further investigated the potential associations between membrane lipid damage and clinical symptoms in adolescents with depression, encompassing depressive symptom severity and cognitive function. WGCNA results showed that five lipid modules were significantly negatively associated with HAMD scores or positively associated with MoCA and WMS scores, which were defined as protection modules (Fig. 4A). In contrast, three lipid modules displayed significant positive correlations with HAMD scores or negative correlations with MoCA and WMS scores, termed as risk modules. The lipid composition characteristics of the protection and risk modules were analyzed by hypergeometric distribution. In terms of lipid category, the protection modules demonstrated significant enrichment in Ether.L, while the risk modules exhibited significant enrichment in Oxid.L and Cer (Fig. 4B and Fig. S5A). As for the fatty acid chain composition, the protection modules predominantly enriched PUFA, while the risk modules significantly enriched SFA (Fig. 4C and Fig. S5B).

Subsequently, we analyzed the relationship between membrane lipid indexes and clinical symptoms by Spearman's rank coefficient (Fig. 4D). The results showed that the n6/n3 PUFA, lipophilic index, and total content of oxidized lipids were significantly negatively correlated with MoCA and WMS scores. PC/SM and peroxidation index were found to be significantly positively correlated with MoCA and WMS scores. These results further suggested that cognitive impairment in adolescents with depression is related to abnormalities in n6/n3 PUFA, membrane fluidity, and oxidative stress. HAMD scores were not significantly associated with any of these membrane lipid indexes (Fig. S6).

### 3.5 Membrane lipid homeostasis was associated with niacin skin flushing response

**Fig. 5:**
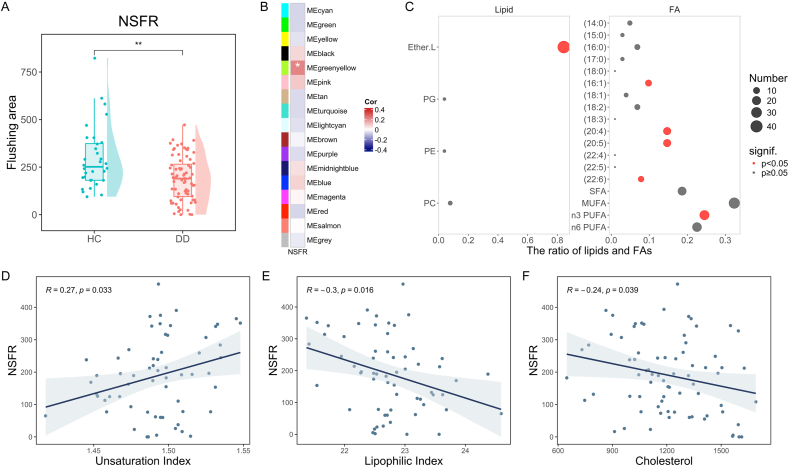
**Membrane lipid homeostasis was associated with niacin skin flushing response of adolescents with depression.** (A) Weakened NSFR in adolescent depression. (B) Correlation between lipid modules and NSFR. ∗p < 0.05. (C) The composition of lipids classes and fatty acids chains in the greenyellow module. The association between NSFR with (D) unsaturation index, (E) lipophilic index, and (F) cholesterol levels. DD, Depressive Disorder; HC, Healthy Control; NSFR, Niacin Skin Flushing Response; PC, Phosphatidylcholine; PE, Phosphatidylethanolamine; PG, Phosphatidylglycerol; Ether.L, Ether Lipid; FA, Fatty Acids; SFA, Saturated Fatty Acids; MUFA, Monounsaturated Fatty Acids; PUFA, Polyunsaturated Fatty Acids.

We proposed that membrane lipid damage is systemic in adolescent depression, which may be reflected in the intensity of NSFR. Our result demonstrated a significant reduction in NSFR in adolescents with depression compared to healthy controls (Fig. 5A). To explore the membrane lipid signatures associated with attenuated NSFR, we identified a lipid module with a significant positive association with NSFR through WGCNA (Fig. 5B). This lipid module was significantly enriched in Ether.L and PUFA, among which AA and EPA accounted for the highest proportion (Fig. 5C). In particular, n3 PUFA was significantly enriched. We further found that NSFR was significantly positively correlated with the unsaturation index (Fig. 5D). In addition, NSFR displayed a significant negative correlation with the lipophilic index (Fig. 5E) and cholesterol levels (Fig. 5F). Taken together, reduced fatty acid unsaturation and fluidity of cell membrane may be closely related to blunted NSFR in depressed adolescents.

## 4 Discussion

**Fig. 6:**
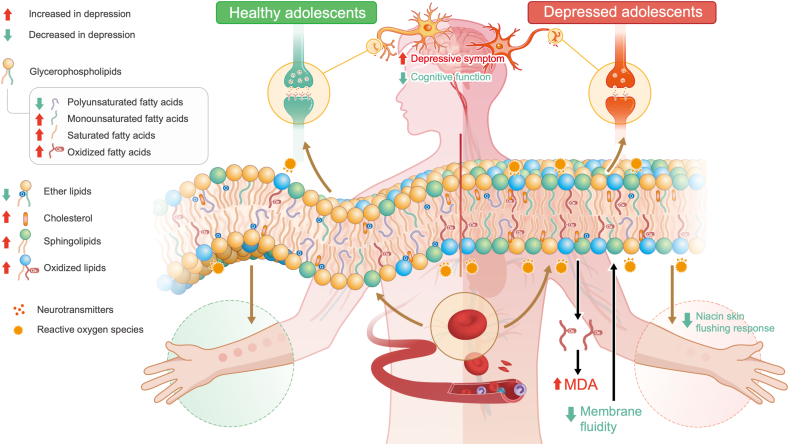
**The disturbance of cell membrane lipid homeostasis was evident in adolescents suffering from depression.** In comparison to healthy controls, depressed adolescents exhibited notable variations in membrane lipid composition, including elevated cholesterol, sphingomyelins, ceramides, and lipid oxidation, as well as reduced ether lipids, polyunsaturated fatty acids, and membrane fluidity. Moreover, membrane lipid damage was found to be associated with depressive symptoms, cognitive function, and niacin skin flushing response in adolescents with depression. These findings suggest that disruptions in membrane lipid composition may have systemic implications and contribute to aberrant neurotransmitter signaling at synapses, thereby playing a role in the onset and progression of adolescent depression. MDA, Malondialdehyde.

To the best of our knowledge, this study represents the first attempt to characterize the erythrocyte membrane lipidome in adolescents with depression, revealing a dysregulation in membrane lipid homeostasis (Fig. 6). Specifically, we observed a reduction in membrane PUFA levels in patients, accompanied by an elevation in SFA and MUFA levels. Furthermore, there was an increase in oxidized lipids, SM, Cer, and cholesterol, along with a decrease in ether lipids. These alterations in membrane lipids were associated with oxidative stress, diminished membrane fluidity, and disrupted lipid rafts, which were further closely linked to depressive symptoms and cognitive function. Moreover, our findings suggested that the attenuated NSFR in adolescent depression could be partially ascribed to the membrane lipid damage.

Adolescents with depression exhibited significant disturbances in membrane fatty acids, characterized by reduced PUFA levels and an elevated n6/n3 PUFA ratio, which may have occurred before the onset of the disease, as similar changes are observed in high-risk individuals for psychiatric disorders [36]. Mendelian randomization analysis yields evidence suggesting that n3 PUFA levels and the degree of unsaturation have a protective causal impact on depression, while the n6/n3 PUFA ratio may contribute a risk causal influence on lifetime depression [37]. Previous studies highlight that the lower dietary intake of n3 PUFA is linked to the higher rates of depression [38]. Several clinical trials have suggested that n3 PUFA supplementation could serve as a potentially effective adjunctive intervention for depression [39,40]. The diverse effects of n3 PUFA on neuronal structure and function encompass anti-inflammatory and antioxidant properties, modulation of neuroendocrine pathways, and activation of crucial neurotransmitters, potentially contributing to depression prevention [[41], [42], [43], [44]]. Concomitant with the decline in PUFA, there was a relative rise in SFA and MUFA levels in adolescent depression. Research has indicated an escalation in PLA2 activity among individuals with depression, which may result in increased hydrolysis of PUFA at the sn2 position of phospholipids [[45], [46], [47]]. Conversely, lysophosphatidylcholine acyltransferases (LPCATs) facilitate the reacylation of fatty acids in membrane. The upregulations of LPCAT1 and LPCAT4 would contribute to increased incorporation of SFA and MUFA into membrane phospholipids due to their substrate preferences for C16:0-CoA and C18:1-CoA, respectively [34]. We speculate that our results of abnormal membrane fatty acid composition were implicative of the perturbation of membrane phospholipid remodeling in depressed adolescents, which needs further validation.

It is reported that PLA2 and LPCAT1/4 activities are conceivably triggered by oxidative stress [34]. Elevated oxidative stress has been noted in adolescent depression [48], which promotes lipid peroxidation and is associated with cell injury. Our investigation unveiled an accumulation of membrane oxidized lipids and raised plasma MDA levels in depressed adolescents, disrupting membrane lipid homeostasis. Pamplona et al. reviewed that elevated lipid oxidation further induces protein damage, which compromises vital cell functions such as energy metabolism and neurotransmission [49]. In addition, studies have proposed that fatty acids shorten in chain length and decrease in unsaturation and peroxidation during oxidative stress [50], which is consistent with our findings. The reduction of fatty acid double bonds is thought to be related to attenuated antioxidant capacity [51], and peroxidation index is negatively associated with lipoxidation-derived protein damage in brain [52]. Moreover, certain lipids and fatty acids with potential antioxidant or pro-oxidative effects were disturbed in adolescents with depression. n3 PUFA is thought to reduce the generation of ROS [53] and lipid peroxidation [54]. While n6/n3 PUFA plays an important role in the regulation of pro-/anti-inflammatory balance and is positively correlated with oxidative stress [48]. Ether lipids have potential antioxidant effects [55] and were reduced in adolescent depression. On the contrary, increased SFA and ceramides levels are thought to aggravate oxidative stress [56,57]. Overall, we propose that oxidative stress leads to lipid damage in cell membranes, which may involve in the pathological mechanisms of adolescent depression.

Oxidative stress is reported to induce augmented membrane rigidity and raft domain numbers [58,59]. The fluidity of cell membrane mainly depends on the content of unsaturated fatty acids, cholesterol levels, and phospholipid species [60], all of which we found to be abnormal in depressed adolescents. The diminished unsaturation of fatty acids encourages the orderly arrangement of the membrane due to more straight hydrophobic tails, resulting in decreased membrane fluidity. Supplementation of PUFA has been shown to increase membrane fluidity [61]. Elevated cholesterol levels in cell membrane could lead to increased membrane rigidity and reduced fluidity [62]. As the PC/SM ratio was reported to be associated with membrane fluidity [63], the reduced PC/SM ratio in our study also suggested a damaged cell deformability in adolescent depression. The decline of fluidity through examining the anisotropy of erythrocyte membranes has been documented in adult depression [64]. Our research extends the findings of reduced membrane fluidity in depressed adolescents from the standpoint of membrane lipid composition. On the other hand, elevated levels of cholesterol and SM may indicate abnormal lipid rafts in adolescent depression. Studies have linked abnormal lipid raft signal transduction to depression, with the regulation of lipid rafts being a target of antidepressant therapies [65,66]. Furthermore, the accumulation of ceramides leads to the reorganization of lipid raft domains on the cell membrane, and pharmacological interventions to reduce ceramide abundance have also shown antidepressant effects [56]. Therefore, our results reveal the presence of abnormal membrane fluidity and lipid rafts in adolescents with depression, which may impact neurotransmission by regulating membrane-bound signal transduction [67].

Our study further found that membrane lipid damage, including decreased PUFA, ether lipids, and membrane fluidity, as well as elevated n6/n3 PUFA and oxidative stress, were associated with more severe depressive symptoms or worse cognitive function in depressed adolescents. The brain is abundant in PUFA, which plays crucial roles in neurogenesis, synaptic function, and the regulation of brain inflammation [68]. Randomized controlled trials in adolescents with depression have demonstrated that n3 PUFA supplementation significantly ameliorated depressive symptoms and cognitive function [40,69], and a higher proportion of EPA in erythrocyte membranes is associated with better cognitive outcomes [70]. In addition, the deficiency of ether lipids is linked to disruptions in synaptic vesicle cycling and reduced neurotransmitter release [71], affecting cognitive, social, and emotional behaviors [72]. The effectiveness of ether lipid supplementation in improving cognition and mobility has been demonstrated in cognitively impaired individuals [73]. On the other hand, both n3 PUFA and ether lipid have certain properties of anti-oxidation and promoting membrane fluidity. The brain is vulnerable to oxidative damage due to its rich lipids, high oxygen utilization, and weaker antioxidative defense [74]. It is supposed that oxidative neuronal injury can be prevented by dietary supplementation of antioxidants [75], which are an effective strategy to treat depression [76]. Moreover, research has observed that patients with mild cognitive impairment exhibit reduced membrane fluidity [77], while the correlation between cognition and fluidity in depressed adolescents is rarely reported. Overall, our findings may provide a molecular basis for developing new treatments to improve the clinical symptoms for adolescent depression.

Finally, we confirmed the attenuation of NSFR in depressed adolescents, which is closely associated with the imbalance of membrane lipid homeostasis. The niacin-induced skin flushing involves multiple steps, including niacin-triggered activation of HCAR2, PLA2-mediated release of AA from the membrane, conversion of free AA to prostaglandins through COX, and subsequent vasodilation induced by prostaglandins [[29], [30], [31], [32]]. The lipid module significantly associated with NSFR exhibited a prominent enrichment in PUFA, a relationship further supported by the positive correlation between NSFR and the unsaturation index. Our previous clinical trial has shown significant enhancement in NSFR after n3 PUFA supplementation in depressed adolescents [40]. Meanwhile, we observed associations between NSFR with lipophilic index, cholesterol, and ether lipids, all of which influence cell membrane fluidity and signal transduction. Therefore, we postulate that membrane lipid damage may impede NSFR in adolescent depression by affecting membrane-protein interactions such as HCAR2 binding [78], PLA2 function [79], or prostaglandins signal [80]. For example, elevated cholesterol levels reduce cyclic adenosine monophosphate signaling across various cellular models [80], a pathway crucial for prostaglandin-induced vasodilation. Moreover, decreased membrane fluidity could lead to reduced cell deformability, consequently diminishing vasodilation capacity [81]. Interestingly, the attenuated NSFR in depressed adolescents may indicate similar membrane impairments as that has been proposed in schizophrenia, which suggests a common pathophysiological mechanism underlying these two mental disorders [34,82]. Therefore, NSFR may be a simple and detectable macroscopic manifestation of membrane lipid damage, further supporting its role as a biomarker for adolescent depression.

There are some limitations in our study. Firstly, the lipid levels reported in erythrocyte membranes may not be fully representative of brain lipid levels. However, previous studies have shown a positive correlation between brain lipid levels and erythrocyte membrane lipids [83,84], indicating that there may be impaired membrane lipid homeostasis in neurons as well. Secondly, while erythrocyte membrane lipids are less influenced by short-term dietary changes compared to plasma or serum lipids [85], adjusting for dietary habits to account for their impact on membrane lipid would enhance the rigor of our study. Thirdly, due to the vast number of lipids, the identification of lipids in our study was not exhaustive, and all membrane lipids were semi-quantified using only seven lipid standards, which were not precise enough. Lastly, our sample was limited to the Han population in southern China. The findings of this study need to be further verified in diverse regions and ethnicities. Moreover, the cross-sectional design of our study limits the ability to infer causality between membrane lipid homeostasis and adolescent depression. Future research, potentially involving animal and longitudinal studies, could further explore how cell membrane lipids contribute to the onset and progression of adolescent depression.

## 5 Conclusion

This study uncovered the membrane lipid damage among depressed adolescents, including reduced membrane fluidity and elevated lipid oxidation, which were strongly associated with depressive symptom, cognitive function, and NSFR. Our research provides support to the phospholipid hypothesis of adolescent depression and sets the groundwork for identifying biomarkers and therapeutic targets.

## CRediT authorship contribution statement

**Jinfeng Wang:** Formal analysis, Investigation, Methodology, Software, Validation, Visualization, Writing – original draft, Writing – review & editing. **Xiaowen Hu:** Methodology, Visualization, Writing – review & editing. **Ya Li:** Methodology, Writing – review & editing. **Shuhui Li:** Methodology, Writing – review & editing. **Tianqi Wang:** Methodology, Writing – review & editing. **Dandan Wang:** Writing – review & editing. **Yan Gao:** Writing – review & editing. **Qian Wang:** Writing – review & editing. **Jiansong Zhou:** Funding acquisition, Supervision, Writing – review & editing. **Chunling Wan:** Conceptualization, Data curation, Funding acquisition, Project administration, Resources, Supervision, Writing – review & editing.

## Funding

This study was supported by the STI2030-Major Projects [grant numbers 2021ZD0200700, 2021ZD0200800], 10.13039/100014717the National Natural Science Foundation of China [grant numbers 82071543, 81971254], 10.13039/100007219the Natural Science Foundation of Shanghai [grant numbers 23ZR1433300], the Key research and development plan of Hunan [grant numbers 2023SK2028], and the Hunan Medical Association [grant numbers HNA202101008].

## Declaration of competing interest

The authors declare that they have no known competing financial interests or personal relationships that could have appeared to influence the work reported in this paper.

## Abbreviations

AA, Arachidonic Acid; AUC, Area Under the Curve; BMI, Body Mass Index; Cer, Ceramide; ChE, Cholesterol Ester; CHO, Cholesterol; COX2, Cyclooxygenase 2; DD, Depressive Disorder; DG, Diglyceride; EPA, Eicosapentaenoic Acid; Ether.L, Ether Lipid; FA, Fatty Acids; FC, Fold Change; FDR, False Discovery Rate; GL, Glycerolipid; GP, Glycerophospholipid; HAMD, Hamilton Depression Scale; HC, Healthy Control; HCAR2, Hydroxycarboxylic Acid Receptor 2; HexCer, Hexosyl Ceramide; ICD-10, International Classification of Diseases, Version 10; LPCATs, Lysophosphatidylcholine Acyltransferases; LPL, Lysophospholipid; LSM, Lysosphingomyelin; MDA, Malondialdehyde; MoCA, Montreal Cognitive Assessment; MUFA, Monounsaturated Fatty Acids; NSFR, Niacin Skin Flushing Response; OPLS-DA, Orthogonal Partial Least Squares-Discriminant Analysis; Oxid.L, Oxidized Lipid; PA, Phosphatidic Acid; PC, Phosphatidylcholine; PCA, Principal Component Analysis; PE, Phosphatidylethanolamine; PG, Phosphatidylglycerol; PGD2, Prostaglandin D2; PGE2, Prostaglandin E2; PI, Phosphatidylinositol; PL, Phospholipid; PLA2, Phospholipase A2; PS, Phosphatidylserine; PUFA, Polyunsaturated Fatty Acids; QC, Quality Control; ROS, Reactive Oxygen Species; SFA, Saturated Fatty Acids; SM, Sphingomyelin; SP, Sphingolipid; ST, Sterol Lipids; TG, Triglyceride; VIP, Variable Importance in the Projection; WGCNA, Weighted Correlation Network Analysis; WMS, Wechsler Memory Scale.
