# Supplementary material for: Impaired lipid homeostasis and elevated lipid oxidation of erythrocyte membrane in adolescent depression
Source: Redox Biol. 2025 Jan 8;80:103491. doi: 10.1016/j.redox.2025.103491 (PMC11780951; doi:10.1016/j.redox.2025.103491)
Supplement: Multimedia component 1 [file mmc1.docx]

**Table S2. The list of 8 lipids in the combinational biomarker panel.**

| Lipid | Class | FDR | FC |
| --- | --- | --- | --- |
| ChE (18:1) | ChE | 3.56E-08 | 0.03 |
| TG (18:4_18:1_18:2) | TG | 4.67E-08 | 1.73 |
| PC (18:1e_20:3) | PC | 1.07E-08 | 0.63 |
| PC (18:2_20:4) | PC | 3.31E-08 | 0.65 |
| PC (22:5_18:2) | PC | 6.99E-08 | 0.59 |
| PS (22:4_18:2) | PS | 3.31E-08 | 0.63 |
| Cer (d16:1_16:1) | Cer | 1.95E-08 | 3.46 |
| Cer (d19:1_24:1) | Cer | 3.31E-08 | 1.50 |

ChE, Cholesterol Ester; TG, Triglyceride; PC, Phosphatidylcholine; PS, Phosphatidylserine; Cer, Ceramide; FDR, False Discovery Rate; FC, Fold Change.


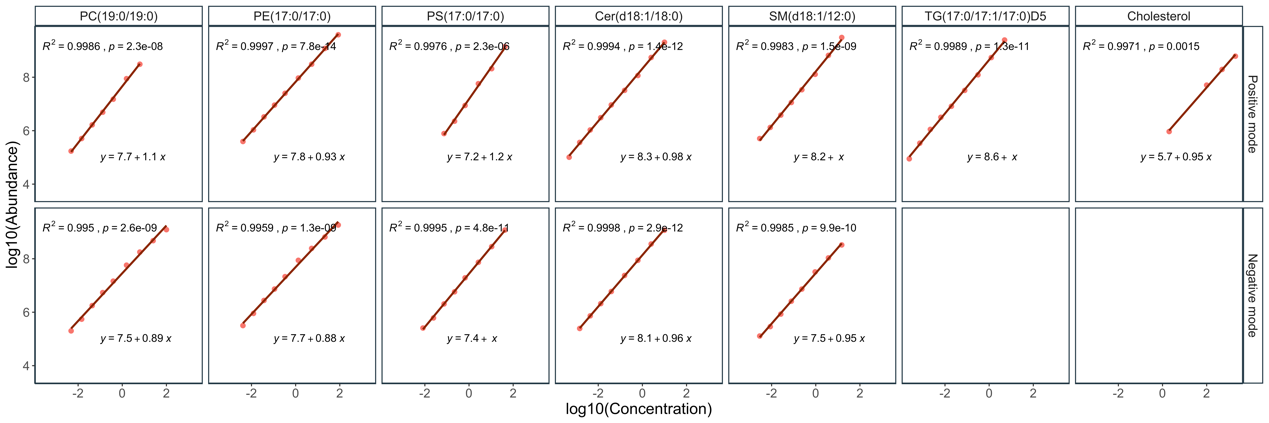


**Figure S1. The external standard curves of seven lipids.** PC, Phosphatidylcholine; PE, Phosphatidylethanolamine; PS, Phosphatidylserine; Cer, Ceramide; SM, Sphingomyelin; TG, Triacylglycerol.


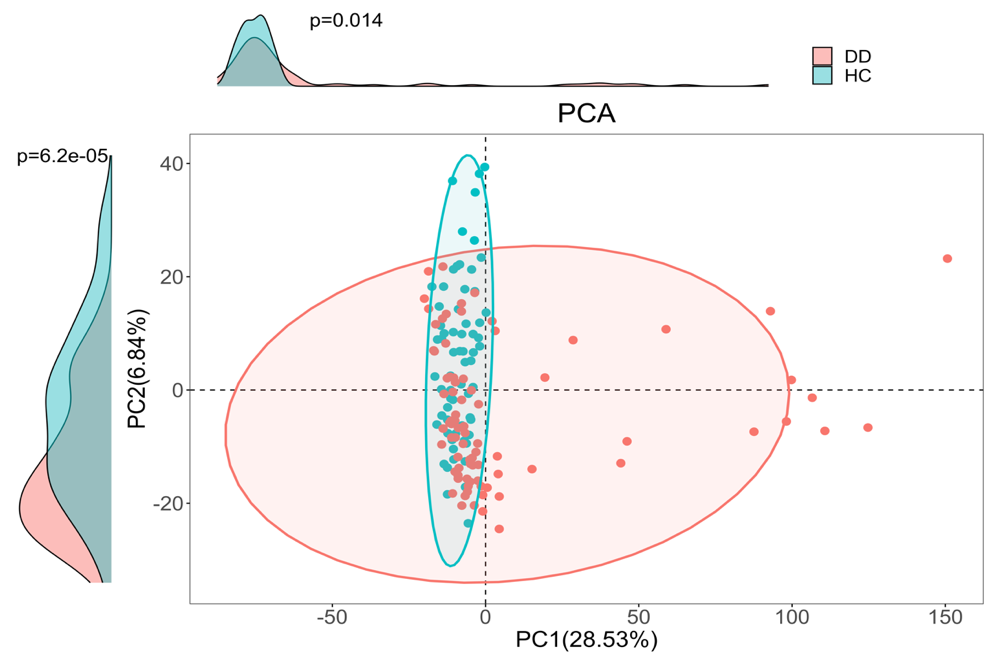


**Figure S2. PCA score plots of erythrocyte membrane lipidomes.** DD, Depressive Disorder; HC, Healthy Control; PCA, Principal Component Analysis.

**
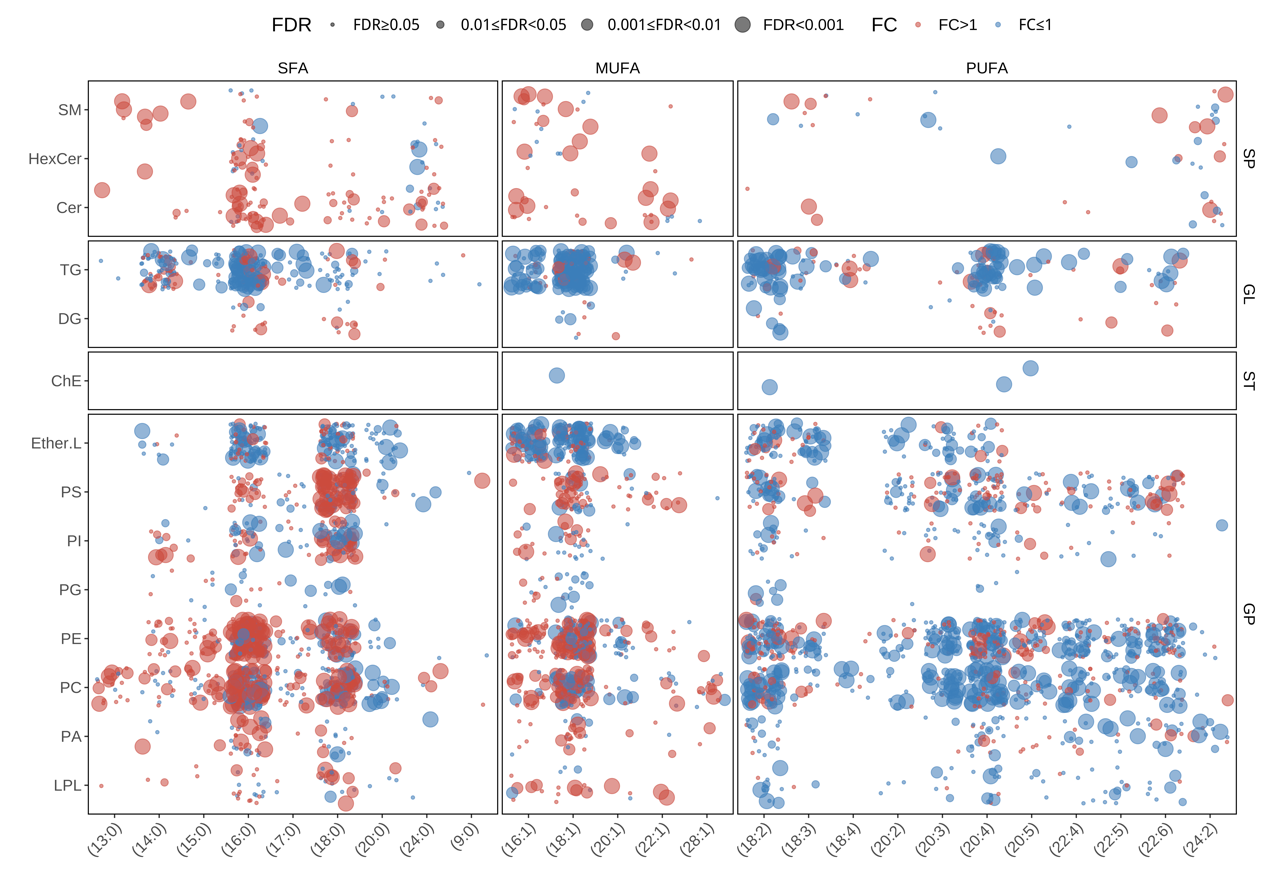
**

**Figure S3.** **The differences of non-oxidized fatty acid chains of each lipid** **between depressed adolescents and controls.** LPL, Lysophospholipid; PA, Phosphatidic Acid; PC, Phosphatidylcholine; PE, Phosphatidylethanolamine; PG, Phosphatidylglycerol; PI, Phosphatidylinositol; PS, Phosphatidylserine; DG, Diglyceride; TG, Triglyceride; Cer, Ceramide; HexCer, Hexosyl Ceramide; SM, Sphingomyelin; ChE, Cholesterol Ester; Ether.L, Ether Lipid; GP, Glycerophospholipid; ST, Sterol Lipids; GL, Glycerolipid; SP, Sphingolipid; FC, Fold Change; FDR, False Discovery Rate.**
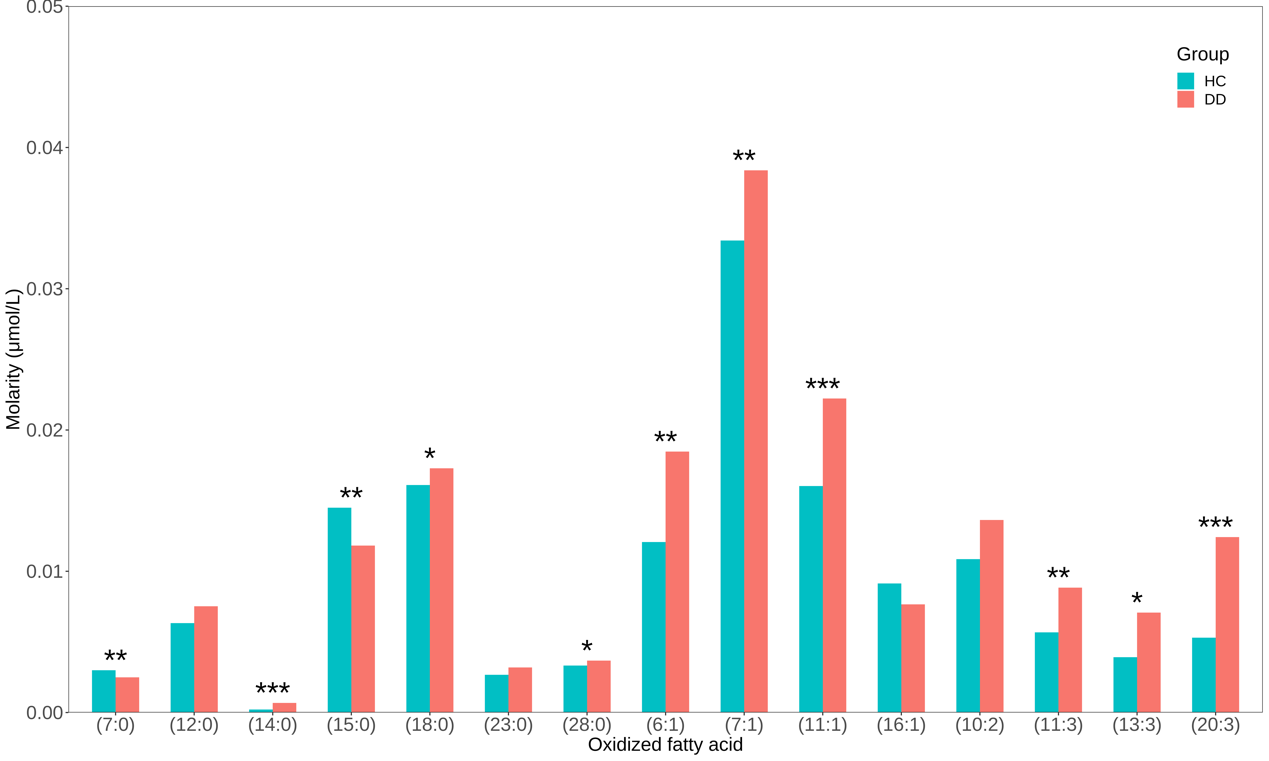
**

**Figure S4. The differences of 15 oxidized fatty acids levels with lower concentrations between depressed adolescents and controls.** DD, Depressive Disorder; HC, Healthy Control. *p<0.05, **p<0.01, ***p<0.001.**
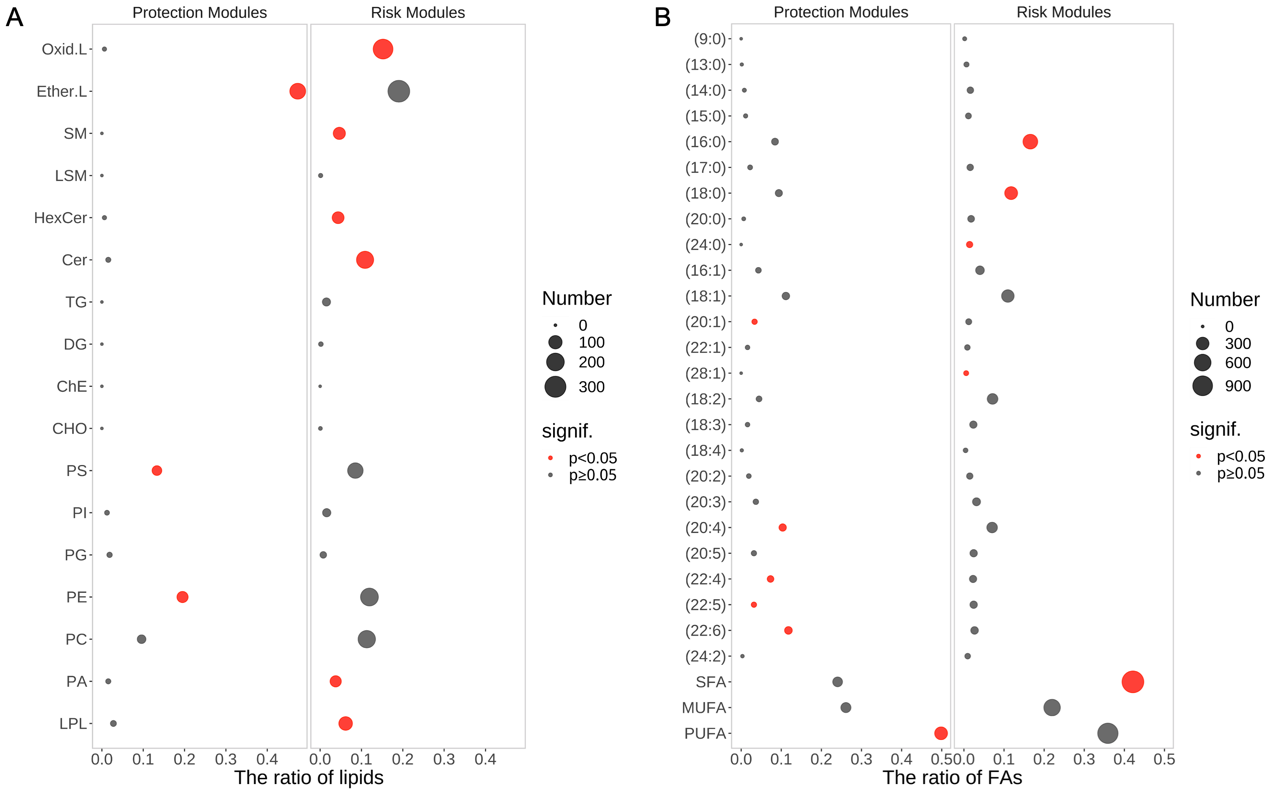
**

**Figure S5. The overall composition of protection and risk modules associated with depressive symptom and cognitive function in adolescents with depression.** (A) Characteristics of different lipid classes. (B) Characteristics of different fatty acid chains. LPL, Lysophospholipid; PA, Phosphatidic Acid; PC, Phosphatidylcholine; PE, Phosphatidylethanolamine; PG, Phosphatidylglycerol; PI, Phosphatidylinositol; PS, Phosphatidylserine; CHO, Cholesterol; ChE, Cholesterol Ester; DG, Diglyceride; TG, Triglyceride; Cer, Ceramide; HexCer, Hexosyl Ceramide; LSM, Lysosphingomyelin; SM, Sphingomyelin; Ether.L, Ether Lipid; Oxid.L, Oxidized Lipids; FA, Fatty Acids; SFA, Saturated Fatty Acids; MUFA, Monounsaturated Fatty Acids; PUFA, Polyunsaturated Fatty Acids.**
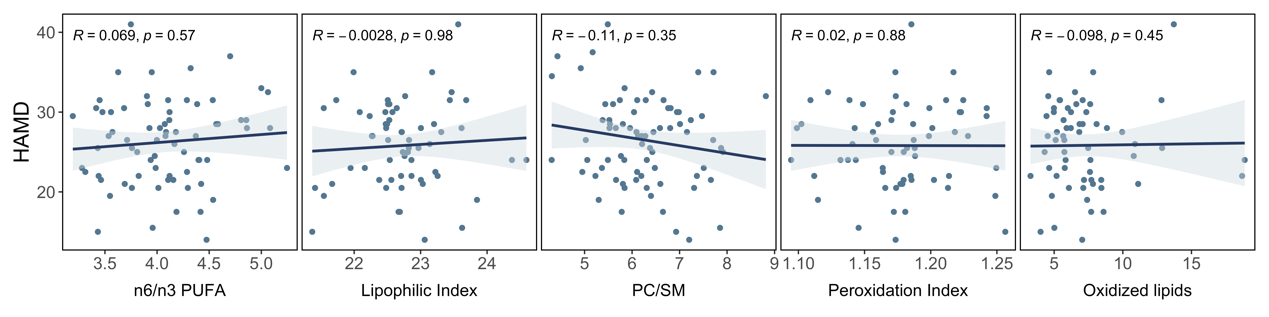
**

**Figure S6.** **The Spearman correlation between HAMD score and n6/n3 PUFA ratio, lipophilic index, PC/SM ratio, peroxidation index and total content of oxidized lipids.** HAMD, Hamilton Depression Scale; PUFA, Polyunsaturated Fatty Acids; PC, Phosphatidylcholine; SM, Sphingomyelin; Oxid.L, Oxidized Lipids.
